# Adherence to the Mediterranean diet regulates the association between osteopenia and the risk of all-cause mortality in general population

**DOI:** 10.1186/s41043-023-00447-6

**Published:** 2023-10-03

**Authors:** Chao Ma, Liangliang Li, Huan Zhao, Jue Zhang

**Affiliations:** 1 The Drum Tower Hospital Affiliated to Medical School of Nanjing University, Nanjing, Jiangsu, 210008 China; 2 Department of Orthopaedics, The Affiliated Jiangning Hospital of Nanjing Medical University, Nanjing, 211100 Jiangsu China; 3 Department of Cardiology, The Affiliated Jiangning Hospital of Nanjing Medical University, Nanjing, 211100 Jiangsu China

**Keywords:** Adherence to the Mediterranean diet, Osteopenia, All-cause mortality, BMD

## Abstract

**Background:**

This study aimed to explore the association of adherence to the Mediterranean diet (MD), osteopenia and the risk of all-cause mortality in general population.

**Methods:**

This retrospective cohort study included 5452 participants ≥ 50 years from the National Health and Nutrition Examination Survey (NHANES). The associations of osteopenia and adherence to the MD with all-cause mortality, as well as the interaction and moderating effects between the osteopenia and adherence to the MD on the all-cause mortality, were explored via univariate and multivariable Cox proportional hazards models.

**Results:**

The follow-up was from October 1, 2006, to December 31, 2019. The median survival time of patients was 81 months. In total, 4724 people were survived and 728 were dead. Osteopenia was associated with increased risk of all-cause mortality in people [hazards ratio (HR) = 1.57, 95% confidence interval (CI) 1.23–1.99]. No significant risk of all-cause mortality was found in people with high adherence to the MD compared with those with low adherence to the MD (*P* > 0.05). Compared to subjects with no osteopenia who had high adherence to the MD, osteopenia people who had high adherence to the MD (HR = 1.52, 95% CI 1.17–1.98) or low adherence to the MD (HR = 1.81, 95% CI 1.23–2.66) were at increased risk of all-cause mortality after adjusting for confounding factors. The relationship between osteopenia and the risk of all-cause mortality was decreased in those with high adherence to the MD (HR = 1.57, 95% CI 1.17–2.11) compared with those with low adherence to the MD (HR = 1.62, 95% CI 1.08–2.41) after adjusting for confounding factors.

**Conclusion:**

The adherence to the MD regulated the association between osteopenia and the risk of all-cause mortality, which suggested the importance of adherence to the MD in those with osteopenia, and the MD could be advocated in general people.

**Supplementary Information:**

The online version contains supplementary material available at 10.1186/s41043-023-00447-6.

## Background

Osteoporosis and osteopenia are characterized by a compromised bone strength and decreased bone mineral density (BMD), which are becoming major health burdens [1, 2]. Osteopenia is considered to be the precursor of osteoporosis, which is defined based on bone densitometry with a T-score -1 to -2.5 according to the World Health Organization [3]. A systematic review and meta-analysis revealed that the prevalence of osteopenia in males was 20.5% compared with 24.4% in females [4]. A previous study indicated that the average lifetime risk of suffering from an osteoporotic fracture was about 40%-50% for women and 13%-22% for men in 50-year-old persons [5]. The risk of fractures is high not only in people with osteoporosis, but also in those with osteopenia. There was evidence revealed that about 50% of the fractures, 50% of the recurrent fractures, and increased risk of morbidity, mortality and costs to the community were found in the population with osteopenia at modest risk for fracture, not the smaller fraction with osteoporosis at high risk for fracture [6]. Also a systematic review and meta-analysis found that BMD level was inversely associated with the all-cause mortality [7]. It is crucial to ascertain the correlation between osteopenia and all-cause mortality within the general population, as this information holds significant importance for managing individuals with osteopenia.

Nutritional support is essential for the prevention and treatment of osteoporosis, and the overall diet quality is more important than individual food components [8]. The Mediterranean diet (MD) is a widely recognized healthy plant-based dietary worldwide as it is mainly based on the traditional foods and drinks, which is associated with better nutrient sufficiency [9]. MD is recommended as one of the healthiest dietary patterns by the 2015–2020 Dietary Guidelines of the USA [10]. Higher adherence to the MD is reported to be associated with higher BMD [11]. Additionally, numerous studies have shown that adherence to the MD was associated with reduced risk of all-cause, cardiovascular disease (CVD), and other specific mortality [12, 13]. Whether there were associations among adherence to the MD, osteopenia and the risk of all-cause mortality in general population were still unclear.

In the present study, the associations among adherence to the MD, osteopenia and the risk of all-cause mortality in general people were explored based on the data from the National Health and Nutrition Examination Survey (NHANES).

## Methods

### Study design and population

This retrospective cohort study included 14,166 individuals ≥ 50 years from the NHANES database. NHANES is conducted by the Centers for Disease Control and Prevention’s National Center for Health Statistics (NCHS) biennially [14]. NHANES is a nationally representative survey assessing the health and nutritional status of the non-institutionalized civilian population in USA through collecting the questionnaire, physical examination data, and biospecimens. The study was approved by the NCHS Research Ethics Review Board, and informed consents were collected from the subjects. In the present study, people without survival information, those without data on BMD, dietary intake (energy, calcium, vitamin D, and dietary supplements), physical activity, and subjects with implausible energy intakes were excluded. Finally, 5,452 participants were included, among them 4,724 people were survived and 728 were dead. The requirement of ethical approval for this was waived by the Institutional Review Board of  The Affiliated Jiangning Hospital of Nanjing Medical University, because the data was accessed from NHANES (a publicly available database). Written informed consent was not required as this study was based on publicly available data. All methods were performed in accordance with the relevant guidelines and regulations.

### Main variables

Osteopenia was evaluated based on the data of BMD. BMD was detected using dual-energy X-ray absorptiometry (DXA) at the femur neck and total femur to calculate the T-score [15]. Osteopenia was defined as femur neck or total femur BMD T-score ≤   − 1.

The MD contains nine food groups (a total of 9 points), and the intakes of the groups were dichotomized by sex-specific median values. When subjects consumed presumed beneficial foods (whole grains, vegetables (excluding potatoes), fruit (including juice), nuts, legumes, fish, and the ratio of monounsaturated fatty acids-to-saturated fatty acids) above the median level and consumed presumed detrimental foods (red and processed meat) below the median level, a score of 1 point was assigned, and 0 point was assigned for all other. A score of 1 was assigned to men who consumed alcohol between 10 and 25 g/day and to women who consumed between 5 and 15 g/day, versus a score of 0 [16, 17].

### Outcome variable

All-cause mortality of subjects was the outcome in this study, and the follow-up was from October 1, 2006, to December 31, 2019. The median survival time of patients was 81 months.

### Potential confounders and definitions

Age (years), gender, race (White or other races), education (high school of above or high school or below), marital status (married or other), poverty-to-income ratio (PIR), drinking (yes or no), smoking (yes or no), metabolic equivalents (METs), hypertension (yes or no), diabetes (yes or no), dyslipidemia (yes or no), CVDs (yes or no), parents previous fracture (yes or no), previous fracture (yes or no), glucocorticoid use (adrenal cortical steroids) (yes or no), anti-osteoporosis therapy (bisphosphonates and miscellaneous bone resorption inhibitors) (yes or no), BMI (kg/m^2^; obesity, overweight, or underweight & normal),waist circumference (cm), total 25-hydroxyvitamin D (mcg), energy (kcal), calcium (mg), vitamin D (mcg), dietary supplements taken (yes or no), hormonetherapy (androgens and anabolic steroids, estrogens, gonadotropins, progestin, sex hormone combinations, miscellaneous hormones, and gonadotropin-releasing hormone and analogs) [inapplicable (male), yes or no], and menopausal [inapplicable (male), yes or no] were potential confounders in the current study.

The PIR was calculated by dividing family (or individual) income to the poverty guidelines specific for each survey year. Physical activity was converted into energy consumption based on the questionnaire in the database. Energy consumption (MET × min) = recommended MET × exercise time of corresponding activity (min), which can be converted into weekly energy consumption. According to the questionnaire, those who smoked at least 100 cigarettes during their lifetime was regarded as smoking. CVD was defined based on the answer of “Yes” to variable MCQ160D (Ever told you had angina or heart failure?), MCQ160E (Ever told you had heart attack?), MCQ160C (Has a doctor or other health professional ever told you that you had coronary heart disease?), MCQ160F (Ever told you had a stroke?), MCQ160B (Ever told had congestive heart failure?), or those received CVD drugs based on 40-CARDIOVASCULAR AGENTS-41, 43, 44, 45, 46, 50, 51, 52, 53, 54, 56, 303, 340, 342, 430, 433, 483. Dyslipidemia was defined based on total cholesterol ≥ 200 mg/dL (5.2 mmol/L) or triglyceride ≥ 150 mg/dL (1.7 mmol/L) or low-density lipoprotein cholesterol ≥ 130 mg/dL (3.4 mmol/L) or high-density lipoprotein cholesterol ≤ 40 mg/dL (1.0 mmol/L), previous physician-diagnosed hypercholesterolemia (BPQ080) or receiving cholesterol-lowering treatment (BPQ090D) or lipid-lowering drugs (358-metabolic agents-19-antihyperlipemic agents). Hypertension was defined as systolic blood pressure ≥ 130 mmHg and or diastolic blood pressure ≥ 80 mmHg or previous physician-diagnosed hypertension (BPQ020) or take blood pressure medications (BPQ040A or drug code 40-CARDIOVASCULAR AGENTS-42, 47, 48, 49, 482, 55). Diabetes was diagnosed based on glycated hemoglobin ≥ 6.5%, fasting glucose ≥ 126 mg/dL, 2 h oral glucose tolerance test blood glucose ≥ 200 mg/dL, previous physician-diagnosed diabetes [DIQ010 (Doctor told you have diabetes)], insulin use (DIQ050) or antidiabetic agents (DIQ070 or 358-metabolic Agents-99-antidiabetic agents). Energy was calculated based on energy intake from Day 1 dietary recall, and the variable in the NHANES database from 1999 to 2002 was DRXTKCAL and from 2003 to 2004 was DR1TKCAL.

### Statistical analysis

Mean ± standard deviation (mean ± SD) was used to describe the measurement data with normal distribution, and t-test was used to compare the differences between the two groups. Median and quartiles [M (Q_1_, Q_3_)] were used to describe the measurement data with abnormal distribution, and Wilcoxon rank sum test was used to compare the differences between the two groups. The number of cases and percentages [n (%)] was used to describe the enumeration data. Chi-square test or Fisher’s exact probability was used to compare the differences between the groups. The weighted univariate Cox proportional hazards model was used to screen out confounding factors. The associations of osteopenia and adherence to the MD with all-cause mortality, and then the interaction and moderating effects between the osteopenia and adherence to the MD were explored via univariate and multivariable Cox proportional hazards models. Model 1 was not adjusted and Model 2 adjusted for confounding factors including age, race, education, marital status, PIR, smoking, hypertension, CVDs, previous fracture, glucocorticoid use, anti-osteoporosis therapy, total 25-hydroxyvitamin D, energy, calcium, BMI, and menopausal. Considering that the application of random forest to fill in data with a missing proportion < 20% may result in bias (Additional file 1: Table S1), sensitivity analysis was conducted by comparing the data before and after interpolation to exclude the influence of random forest interpolation on the study results, and no significant difference was observed (Additional file 1: Table S2). Subgroup analysis was conducted in people in different gender, age, BMI groups and those with or without CVDs. Hazards ratio (HR) and 95% confidence interval (CI) were employed for evaluating the associations among adherence to the MD, osteopenia and the risk of all-cause mortality in general population. Missing value interpolation was performed using Python 3.7.4. Sensitivity analysis and difference comparisons were performed by SAS 9.4 (SAS Institute Inc., Cary, NC, USA). The weighted univariate/multivariate Cox proportional hazards model modeling and subgroup analysis were completed by R version 4.2.0 (2022–04-22 ucrt).

## Results

### The baseline characteristics of participants

**Fig. 1:**
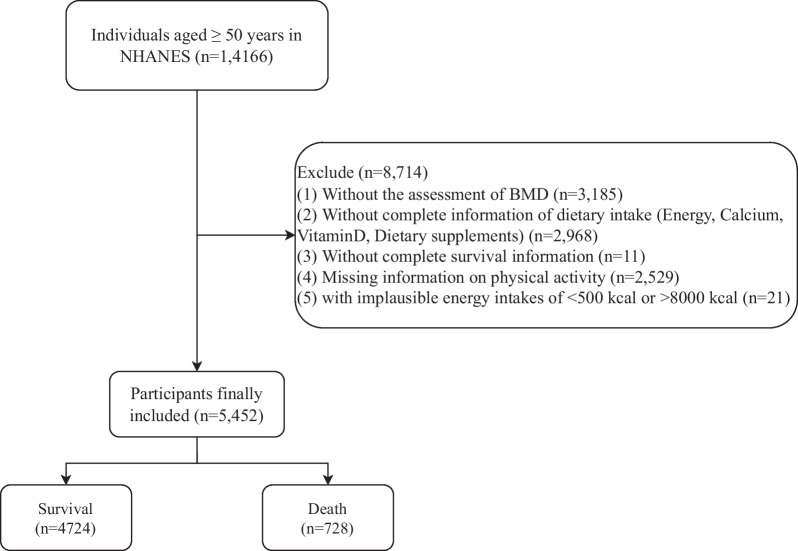
The flow chart of participants’ screen process

As the data of adherence to the MD were measured during 2005–2018 and BMD at the femur neck and total femur was measured during 2005–2006, 2007–2008, 2009–2010, 2013–2014 and 2017–2018, the samples were collected from 2005–2006, 2007–2008, 2009–2010, 2013–2014 and 2017–2018. In total, the data of 14,166 individuals ≥ 50 years were extracted from NHANES database. Among them, those without BMD data (*n* = 3,185), dietary intake data (*n* = 2,968), survival information (*n* = 11), physical activity data (*n* = 2,529), and those with implausible energy intakes (*n* = 21) were excluded. Finally, 5,452 participants were included, among them 4,724 people were survived and 728 were dead. The screen process of participants is shown in Fig. 1.

The percentages of participants who were < 65 years (64.19% vs 25.41%), and married (62.49% vs 50.14%) in the survival group were higher than the death group. The proportion of subjects who had hypertension (58.87% vs 71.70%), CVDs (16.09% vs 36.40%), previous fracture (1.29% vs 2.75%) and anti-osteoporosis therapy (2.94% vs 4.95%) in the survival group were lower than the death group. The median energy intake in the survival group was higher than the death group (1847.25 kcal vs 1769.00 kcal). The percentage of female subjects who were menopausal was higher in the survival group than the death group (42.00% vs 33.10%). The percentage of people with osteopenia in the survival group was lower than the death group (26.99% vs 39.01%). The percentage of people with MED ≥ 4 in the survival group was higher than the death group (67.87% vs 62.77%) (Table 1).

**Table 1:** Comparisons of baseline characteristics of participants survived and dead

Variables	Total (*n* = 5452)	All-cause death		Statistics	*P*
		No (*n* = 4724)	Yes (*n* = 728)		
Age, years, n (%)				χ^2^ = 348.740	< 0.001
< 65	3123 (57.28)	2938 (62.19)	185 (25.41)		
≥ 65	2329 (42.72)	1786 (37.81)	543 (74.59)		
Gender, n (%)				χ^2^ = 40.384	< 0.001
Female	2513 (46.09)	2257 (47.78)	256 (35.16)		
Male	2939 (53.91)	2467 (52.22)	472 (64.84)		
Race, n (%)				χ^2^ = 115.632	< 0.001
Other	2651 (48.62)	2432 (51.48)	219 (30.08)		
White	2801 (51.38)	2292 (48.52)	509 (69.92)		
Education, n (%)				χ^2^ = 41.367	< 0.001
High School of above	2991 (54.86)	2672 (56.56)	319 (43.82)		
High School or below	2461 (45.14)	2052 (43.44)	409 (56.18)		
Marital status, n (%)				χ^2^ = 40.395	< 0.001
Married	3317 (60.84)	2952 (62.49)	365 (50.14)		
Other	2135 (39.16)	1772 (37.51)	363 (49.86)		
PIR, M (Q_1_,Q_3_)	2.64 (1.46, 4.62)	2.77 (1.51, 4.81)	2.06 (1.20, 3.33)	Z = − 8.981	< 0.001
Drinking, n (%)				χ^2^ = 0.014	0.906
No	1473 (27.02)	1275 (26.99)	198 (27.20)		
Yes	3979 (72.98)	3449 (73.01)	530 (72.80)		
Smoking, n (%)				χ^2^ = 48.821	< 0.001
No	2754 (50.51)	2474 (52.37)	280 (38.46)		
Yes	2698 (49.49)	2250 (47.63)	448 (61.54)		
METs, M (Q_1_, Q_3_)	480.00 (200.00, 1200.00)	480.00 (230.00, 1200.00)	300.00 (120.00, 960.00)	Z = − 6.424	< 0.001
Hypertension, n (%)				χ^2^ = 43.507	< 0.001
No	2149 (39.42)	1943 (41.13)	206 (28.30)		
Yes	3303 (60.58)	2781 (58.87)	522 (71.70)		
Diabetes, n (%)				χ^2^ = 0.439	0.508
No	2203 (40.41)	1917 (40.58)	286 (39.29)		
Yes	3249 (59.59)	2807 (59.42)	442 (60.71)		
Dyslipidemia, n (%)				χ^2^ = 0.308	0.579
No	900 (16.51)	785 (16.62)	115 (15.80)		
Yes	4552 (83.49)	3939 (83.38)	613 (84.20)		
CVDs, n (%)				χ^2^ = 170.496	< 0.001
No	4427 (81.20)	3964 (83.91)	463 (63.60)		
Yes	1025 (18.80)	760 (16.09)	265 (36.40)		
Parents previous fracture, n (%)				χ^2^ = 1.707	0.191
No	4876 (89.44)	4235 (89.65)	641 (88.05)		
Yes	576 (10.56)	489 (10.35)	87 (11.95)		
Previous fracture, n (%)				χ^2^ = 9.136	0.003
No	5371 (98.51)	4663 (98.71)	708 (97.25)		
Yes	81 (1.49)	61 (1.29)	20 (2.75)		
Glucocorticoid use, n (%)				χ^2^ = 19.283	< 0.001
No	5328 (97.73)	4633 (98.07)	695 (95.47)		
Yes	124 (2.27)	91 (1.93)	33 (4.53)		
Anti-osteoporosis therapy, n (%)				χ^2^ = 8.143	0.004
No	5277 (96.79)	4585 (97.06)	692 (95.05)		
Yes	175 (3.21)	139 (2.94)	36 (4.95)		
BMI, kg/m^2^, n (%)				χ^2^ = 21.355	< 0.001
Obesity	1937 (35.53)	1716 (36.33)	221 (30.36)		
Overweight	2098 (38.48)	1829 (38.72)	269 (36.95)		
Underweight & Normal	1417 (25.99)	1179 (24.96)	238 (32.69)		
Waist circumference, cm, Mean ± SD	100.35 ± 13.72	100.37 ± 13.61	100.20 ± 14.45	t = 0.30	0.767
Total 25 Hydroxyvitamin D, mcg, M (Q_1_, Q_3_)	69.50 (53.30, 88.16)	69.90 (53.45, 88.65)	67.80 (52.60, 84.58)	Z = − 2.181	0.029
Energy, kcal, M (Q_1_, Q_3_)	1834.75 (1417.75, 2358.50)	1847.25 (1425.50, 2379.75)	1769.00 (1380.00, 2232.00)	Z = − 3.150	0.002
Calcium, mg, M (Q_1_, Q_3_)	806.50 (575.25, 1100.25)	808.50 (579.50, 1101.25)	796.00 (556.25, 1092.75)	Z = − 0.789	0.430
Vitamin D, mcg, M (Q_1_, Q_3_)	3.70 (2.00, 6.10)	3.65 (2.00, 6.05)	3.95 (2.23, 6.50)	Z = 2.773	0.006
Dietary supplements taken, n (%)				χ^2^ = 2.970	0.085
Yes	3525 (64.66)	3075 (65.09)	450 (61.81)		
No	1927 (35.34)	1649 (34.91)	278 (38.19)		
Menopausal, n (%)				χ^2^ = 47.352	< 0.001
Inapplicable (Male)	2939 (53.91)	2467 (52.22)	472 (64.84)		
No	288 (5.28)	273 (5.78)	15 (2.06)		
Yes	2225 (40.81)	1984 (42.00)	241 (33.10)		
Hormonetherapy, n (%)				χ^2^ = 40.637	< 0.001
Yes	175 (3.21)	160 (3.39)	15 (2.06)		
No	2367 (43.42)	2122 (44.92)	245 (33.65)		
Inapplicable	2910 (53.37)	2442 (51.69)	468 (64.29)		
Osteopenia, n (%)				χ^2^ = 44.644	< 0.001
No	3893 (71.40)	3449 (73.01)	444 (60.99)		
Yes	1559 (28.60)	1275 (26.99)	284 (39.01)		
MED, n (%)				χ^2^ = 7.417	0.006
< 4	1789 (32.81)	1518 (32.13)	271 (37.23)		
≥ 4	3663 (67.19)	3206 (67.87)	457 (62.77)		
Time, months, M (Q_1_, Q_3_)	81.00 (35.00, 128.00)	82.00 (34.00, 130.00)	72.00 (39.00, 103.00)	Z = − 9.348	< 0.001

*SD* standard deviation, *M* Median, *Q*_1_ 1st Quartile, *Q*_3_ 3st Quartile, *PIR* poverty-to-income ratio, *METs* metabolic equivalents, *CVDs* cardiovascular diseases, *BMI* body mass index, *MED* Mediterranean diet

### Potential confounding factors for the risk of all-cause mortality in people

**Fig. 2:**
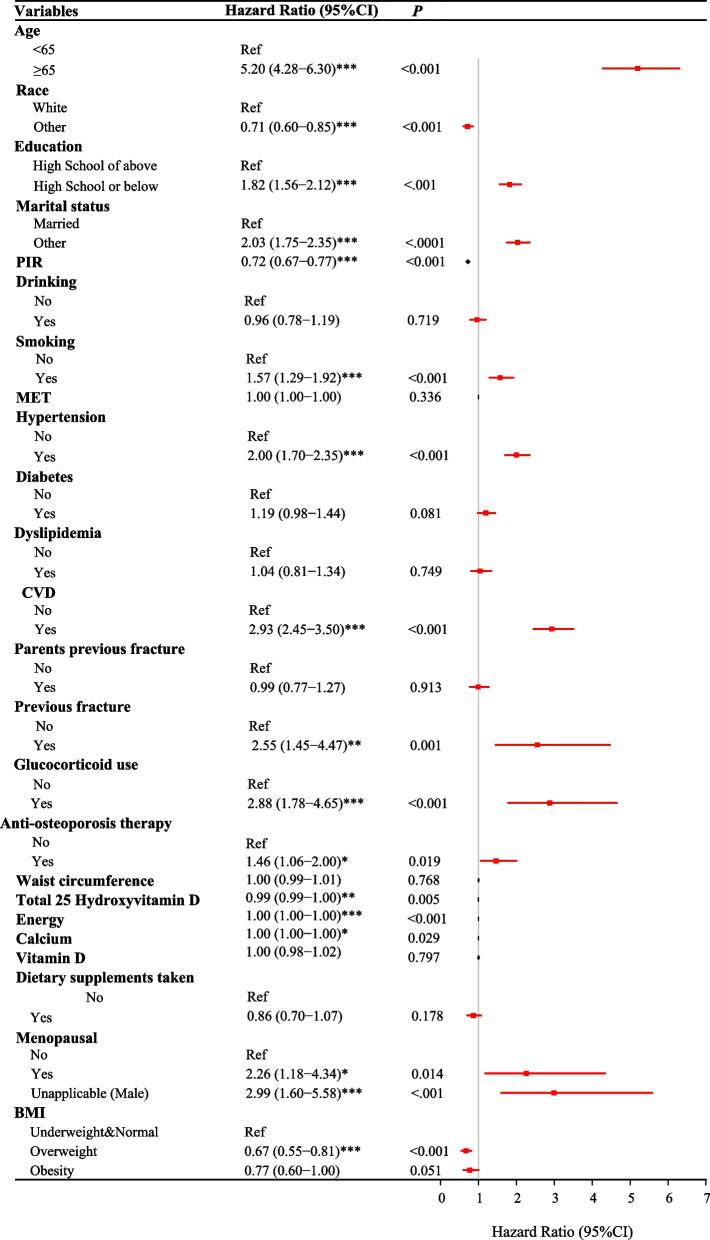
Forest plot revealing the potential confounding factors for the risk of all-cause mortality in people

As observed in Fig. 2, age > 65 years (HR = 5.20, 95% CI 4.28–6.30), high school or below education (HR = 1.82, 95% CI 1.56–2.12), non-married (HR = 2.03, 95% CI 1.75–2.35), smoking (HR = 1.57, 95% CI 1.29–1.92), hypertension (HR = 2.00, 95% CI 1.70–2.35), CVDs (HR = 2.93, 95% CI 2.45–3.50), previous fracture (HR = 2.55, 95% CI 1.45–4.47), glucocorticoid use (HR = 2.88, 95% CI 1.78–4.65), anti-osteoporosis therapy (HR = 1.46, 95% CI 1.06–2.00), and menopausal (HR = 2.26, 95% CI 1.18–4.34) were associated with increased risk of all-cause mortality in people. Non-White races (HR = 0.71, 95% CI 0.60–0.85) and overweight (HR = 0.67, 95% CI 0.55–0.81) were correlated with decreased risk of all-cause mortality in general population.

### Interaction effects of adherence to the MD and osteopenia on the risk of all-cause mortality in general population

**Fig. 3:**
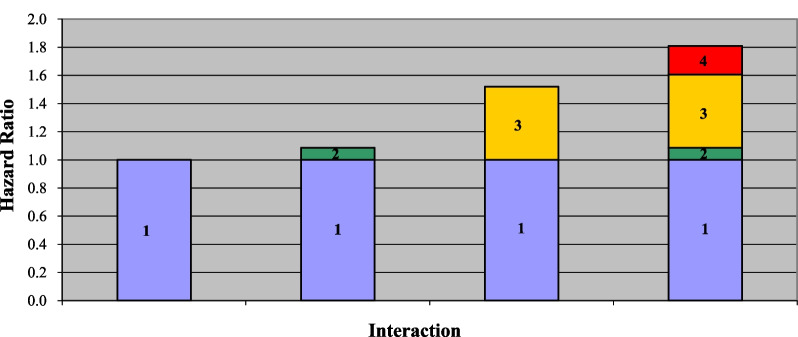
Histogram showing the HRs of adherence to the MD and osteopenia on the risk of all-cause mortality in general population. 1: no osteopenia and high adherence to the MD group, 2: no osteopenia and low adherence to the MD group, 3: osteopenia and high adherence to the MD group, and 4: osteopenia and low adherence to the MD group

Whether adherence to the MD and osteopenia had interaction effects on the risk of all-cause mortality in general population was evaluated, and the results were exhibited in Table 2. The data revealed that osteopenia was associated with increased risk of all-cause mortality in people (HR = 1.57, 95% CI 1.23–1.99). No significant risk of all-cause mortality was found in people with high adherence to the MD compared with those with low adherence to the MD (*P* > 0.05). Compared with no osteopenia subjects who had high adherence to the MD, osteopenia people who had high adherence to the MD (HR = 1.52, 95% CI 1.17–1.98) or low adherence to the MD (HR = 1.81, 95% CI 1.23–2.66) were associated with increased risk of all-cause mortality after adjusting for confounding factors including age, race, education, marital status, PIR, smoking, hypertension, CVDs, previous fracture, glucocorticoid use, anti-osteoporosis therapy, total 25 hydroxyvitamin D, energy, calcium, BMI, and menopausal. Although no interaction effect of adherence to the MD and osteopenia on the risk of all-cause mortality in general population was observed, the HR of people with osteopenia and was higher than those with no osteopenia and high adherence to the MD, also those with osteopenia and low adherence to the MD had higher HRs for the risk of all-cause mortality than those with no osteopenia and high adherence to the MD (Fig. 3).

**Table 2:** Interaction effects of adherence to the MD and osteopenia on the risk of all-cause mortality in general population

Variables	Number	Model 1		Model 2	
		HR (95% CI)	*P*	HR (95% CI)	*P*
Osteopenia					
No	3893	1.00 (Ref)		1.00 (Ref)	
Yes	1559	1.77 (1.43–2.20)	< 0.001	1.57 (1.23–1.99)	< 0.001
Adherence to the MD					
Low	1789	1.00 (Ref)		1.00 (Ref)	
High	3663	0.85 (0.69–1.04)	0.112	0.89 (0.71–1.11)	0.298
Interaction					
No: High	2627	1.00 (Ref)		1.00 (Ref)	
No: Low	1266	1.15 (0.90–1.48)	0.264	1.09 (0.85–1.39)	0.512
Yes: High	1036	1.73 (1.34–2.24)	< 0.001	1.52 (1.17–1.98)	0.002
Yes: Low	523	2.13 (1.53–2.97)	< 0.001	1.81 (1.23–2.66)	0.003

*Ref* reference, *HR* hazard ratio, *CI* confidence Interval, *MD* Mediterranean diet

Model 1: Unadjusted model

Model 2 Adjusted for age, race, education, marital status, PIR, smoking, hypertension, CVDs, previous fracture, glucocorticoid use, anti-osteoporosis therapy, total 25 Hydroxyvitamin D, energy, calcium, BMI, and menopausal

### Adherence to the MD regulating the association between osteopenia and the risk of all-cause mortality in general population

**Fig. 4:**
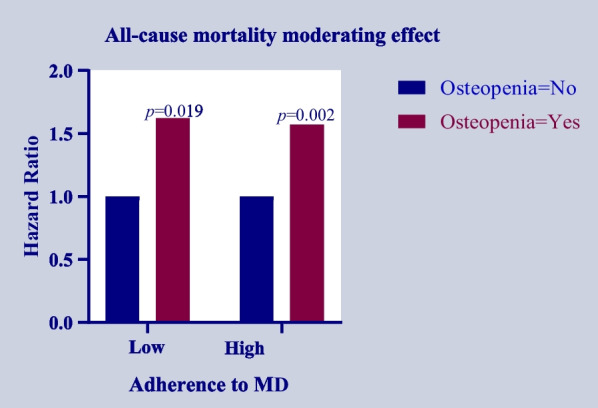
Histogram depicting the HRs of MD on the association between osteopenia and the risk of all-cause mortality in general population

To further explore the role of adherence to the MD on the association between osteopenia and the risk of all-cause mortality in general population, adherence to the MD was regarded as regulating factor, and divided into the high adherence to the MD group and the low adherence to the MD group in the weighted multivariable cox proportional hazards model. The results depicted that the relationship between osteopenia and the risk of all-cause mortality was decreased in those with high adherence to the MD (HR = 1.57, 95% CI 1.17–2.11) compared with those with low adherence to the MD (HR = 1.62, 95% CI 1.08–2.41) after the adjustment of confounding factors including age, race, education, marital status, PIR, smoking, hypertension, CVDs, previous fracture, glucocorticoid use, anti-osteoporosis therapy, total 25 hydroxyvitamin D, energy, calcium, BMI, and menopausal (Table 3, Fig. 4).

**Table 3:** Adherence to the MD in relation to osteopenia and the risk of all-cause mortality in general population

Variables		Osteopenia = No		Osteopenia = Yes	
Adherence to the MD	Model	HR (95% CI)	*P*	HR (95% CI)	*P*
Low	Model 1	1.00 (Ref)		1.85 (1.33–2.58)	< 0.001
	Model 2	1.00 (Ref)		1.62 (1.08–2.41)	0.019
High	Model 1	1.00 (Ref)		1.73 (1.34–2.24)	< 0.001
	Model 2	1.00 (Ref)		1.57 (1.17–2.11)	0.002

*Ref* reference, *HR* hazard ratio, *CI* confidence Interval, *MD* Mediterranean diet

Model 1: Unadjusted model

Model 2 Adjusted for age, race, education, marital status, PIR, smoking, hypertension, CVDs, previous fracture, glucocorticoid use, anti-osteoporosis therapy, total 25 Hydroxyvitamin D, energy, calcium, BMI, and menopausal

### Subgroup analysis of the regulating effect of adherence to the MD on the association between osteopenia and the risk of all-cause mortality in general population

In comparison with people with no osteopenia and high adherence to the MD, increased risk of all-cause mortality was identified in those with osteopenia and low adherence to the MD (HR = 1.99, 95% CI 1.36–2.91) as well as those with osteopenia and high adherence to the MD (HR = 1.64, 95% CI 1.24–2.15) in people ≥ 65 years. In males, increased risk of all-cause mortality was found in those with osteopenia and low adherence to the MD (HR = 2.14, 95% CI 1.31–3.50) as well as those with osteopenia and high adherence to the MD (HR = 1.52, 95% CI 1.07–2.17) compared with people with no osteopenia and high adherence to the MD. In people who were underweight and those with normal BMI, we observed that the risk of all-cause mortality was increased in those with osteopenia and low adherence to the MD (HR = 1.96, 95% CI 1.05–3.64) as well as those with osteopenia and high adherence to the MD (HR = 1.65, 95% CI 1.01–2.70). In subjects without CVDs, increased risk of all-cause mortality was observed in people with osteopenia and low adherence to the MD (HR = 1.80, 95% CI 1.20–2.70) as well as those with osteopenia and high adherence to the MD (HR = 1.46, 95% CI 1.01–2.11). In people complicated with CVDs, we found elevated risk of all-cause mortality in people without osteopenia and low adherence to the MD (HR = 1.47, 95% CI 1.09–1.98), with osteopenia and low adherence to the MD (HR = 1.83, 95% CI 1.08–3.10) and those with osteopenia and high adherence to the MD (HR = 1.78, 95% CI 1.19–2.66) (Table 4).

**Table 4:** Subgroup analysis of the regulating effect of adherence to the MD on the association between osteopenia and the risk of all-cause mortality in general population

Subgroup	Variables	Osteopenia = No		Osteopenia = Yes	
	Adherence to the MD	HR (95% CI)	*P*	HR (95% CI)	*P*
Age					
< 65 (*n* = 3123)	Low	0.91 (0.58–1.44)	0.690	1.49 (0.64–3.45)	0.350
	High	1.00 (Ref)		1.21 (0.74–1.97)	0.451
≥ 65 (*n* = 2329)	Low	1.19 (0.92–1.55)	0.178	1.99 (1.36–2.91)	< 0.001
	High	1.00 (Ref)		1.64 (1.24–2.15)	< 0.001
Gender					
Male (*n* = 2939)	Low	1.07 (0.83–1.38)	0.610	2.14 (1.31–3.50)	0.002
	High	1.00 (Ref)		1.52 (1.07–2.17)	0.020
Female (* n* = 2513)	Low	1.14 (0.59–2.22)	0.690	1.61 (0.97–2.67)	0.066
	High	1.00 (Ref)		1.41 (0.94–2.13)	0.100
BMI					
Underweight & Normal (*n* = 1417)	Low	1.55 (0.93–2.57)	0.092	1.96 (1.05–3.64)	0.034
	High	1.00 (Ref)		1.65 (1.01–2.70)	0.045
Overweight (*n* = 2098)	Low	0.98 (0.70–1.38)	0.922	1.73 (0.91–3.28)	0.095
	High	1.00 (Ref)		1.36 (0.93–1.99)	0.115
Obesity (*n* = 1937)	Low	0.93 (0.61–1.42)	0.743	1.60 (0.93–2.76)	0.091
	High	1.00 (Ref)		1.51 (0.85–2.68)	0.163
CVDs					
No (*n* = 4427)	Low	0.91 (0.63–1.32)	0.618	1.80 (1.20–2.70)	0.005
	High	1.00 (Ref)		1.46 (1.01–2.11)	0.042
Yes (*n* = 1025)	Low	1.47 (1.09–1.98)	0.011	1.83 (1.08–3.10)	0.025
	High	1.00 (Ref)		1.78 (1.19–2.66)	0.005

*Ref* reference, *HR* hazard ratio, *CI* confidence Interval, *MD* Mediterranean diet, *BMI* body mass index, *CVDs* cardiovascular diseases

Multivariable model, if not stratified, adjusted for age, race, education, marital status, PIR, smoking, hypertension, CVDs, previous fracture, glucocorticoid use, anti-osteoporosis therapy, total 25 Hydroxyvitamin D, energy, calcium, and BMI

In female subgroup, menopausal and hormonetherapy were also adjusted

## Discussion

The present study evaluated the associations among adherence to the MD, osteopenia, and the risk of all-cause mortality in general population using the data from NHANES database. The results delineated that osteopenia was associated with increased risk of all-cause mortality in general population. The risk of all-cause mortality in people with osteopenia was decreased in those with high adherence to the MD compared with low adherence to the MD. Subgroup analysis revealed that the risk of all-cause mortality in people with osteopenia was decreased in those with high adherence to the MD compared with low adherence to the MD in subjects ≥ 65 years, males, and people underweight or normal weight. The findings of our study might provide a reference for encouraging more people to have high adherence to the MD in people with osteopenia.

BMD indicates the amount of bone mineral in bone tissue, which is an essential biomarker for the assessment of osteopenia and osteoporosis [18]. Previously, there was evidence showed that BMD was associated with the risk of vascular calcification, and could be regarded as a surrogate marker for vascular events and mortality [19, 20]. BMD constitutes a novel imaging marker that recently has been shown to provide valuable prognostic insights for various severe illnesses including CVDs and chronic lung pathologies [7, 21]. Another study found that low BMD was linked with increased risk of mortality in women with type 2 diabetes [22]. These studies might provide support for the findings in our study. Herein, we found osteopenia, a state of low BMD, was associated with elevated risk of all-cause mortality in general population. Another important finding in this study was that the risk of all-cause mortality in people with osteopenia was decreased in those with high adherence to the MD compared with low adherence to the MD, which suggested that adherence to the MD played a role in the association between osteopenia and all-cause mortality in general population. MD contains nitrate-rich green leafy vegetables, nutrition and so on, which affects multiple biological processes such as protein synthesis and oxidative stress [23]. MD was reported to have a significant effect on regulating the levels of the inflammatory biomarkers such as interleukin-6 and C-reactive protein [24]. Higher adherence to the MD might decrease inflammation in people with osteopenia and then regulate the association between osteopenia and the risk of all-cause mortality in general population. The findings reminded that MD was advocated in general population, and strategies for helping people with osteopenia increase the adherence to the MD were required and could be popularized. The results from subgroup analysis suggested that for old people, males, and underweight or normal weight people with osteopenia, increasing the adherence to the MD might be essential for improving of overall prognosis.

This study assessed the association among adherence to the MD, osteopenia, and the risk of all-cause mortality in general population using the data from NHANES database, which suggested the importance of adherence to the MD in osteopenia people. NHANES data is representative of the non-institutionalized civilian population in USA, and the results might be generalized to the whole USA. The survival information was obtained from the death certificate, and the outcome assessment was accurate, and the loss to follow-up rate was low. Several limitations existed in the present study. Firstly, only dietary intake data were collected at baseline, which may not reflect long-term dietary patterns and changes during follow-up. We used the average of two 24-h dietary recall interviews, which could better reflect the dietary patterns of subjects during that period of time. Secondly, although confounders such as socioeconomic factors, comorbidities, and treatment were adjusted, other potential confounders such as disease status and treatments during follow-up were not adjusted. Thirdly, the doses of drugs used were not reported in the NHANES database. In the future, more well-designed studies were required to verify the results in this study.

## Conclusion

The association among adherence to the MD, osteopenia and the risk of all-cause mortality in general people was explored in the present study. We found that osteopenia was associated with the risk of all-cause mortality, and adherence to the MD regulated the association between osteopenia and the risk of all-cause mortality. The results suggested the importance of adherence to the MD in those with osteopenia, and MED could be advocated in general people.

### Supplementary Information

**Additional file 1**. **Table S1.** The numbers and percentages of missing values. **Table S2.** Sensitivity analysis of missing values manipulation.

## Data Availability

The datasets generated and/or analyzed during the current study are available in the NHANES database, https://wwwn.cdc.gov/nchs/nhanes/.

## Abbreviations

BMD: Bone mineral density
MD: Mediterranean diet
aMED: Adherence to the MED
NHANES: National Health and Nutrition Examination Survey
BMI: Body mass index
CVDs: Cardiovascular diseases
NCHS: National Center for Health Statistics
PIR: Poverty-to-income ratio
METs: Metabolic equivalents
DXA: Dual-energy X-ray absorptiometry

## References

[1] Hauger AV, Bergland A, Holvik K, Ståhle A, Emaus N, Strand BH (2018). Osteoporosis and osteopenia in the distal forearm predict all-cause mortality independent of grip strength: 22-year follow-up in the population-based Tromsø Study. Osteoporosis Int.

[2] Kanis JA. Assessment of fracture risk and its application to screening for postmenopausal osteoporosis: synopsis of a WHO report. WHO Study Group. Osteoporosis international : a journal established as result of cooperation between the European Foundation for Osteoporosis and the National Osteoporosis Foundation of the USA. 1994; 4: 368–81. 10.1007/BF01622200 7696835

[3] Zhang S, Huang X, Zhao X, Li B, Cai Y, Liang X (2022). Effect of exercise on bone mineral density among patients with osteoporosis and osteopenia: a systematic review and network meta-analysis. J Clin Nurs.

[4] Zamani M, Zamani V, Heidari B, Parsian H, Esmaeilnejad-Ganji SM (2018). Prevalence of osteoporosis with the World Health Organization diagnostic criteria in the Eastern Mediterranean Region: a systematic review and meta-analysis. Arch Osteoporos.

[5] Johnell O, Kanis J (2005). Epidemiology of osteoporotic fractures. Osteoporosis Int.

[6] Bala Y, Zebaze R, Ghasem-Zadeh A, Atkinson EJ, Iuliano S, Peterson JM (2014). Cortical porosity identifies women with osteopenia at increased risk for forearm fractures. J Bone Miner Res.

[7] Qu X, Huang X, Jin F, Wang H, Hao Y, Tang T (2013). Bone mineral density and all-cause, cardiovascular and stroke mortality: a meta-analysis of prospective cohort studies. Int J Cardiol.

[8] Chen LR, Hou PH, Chen KH (2019). Nutritional support and physical modalities for people with osteoporosis: current opinion. Nutrients.

[9] Dominguez LJ, Di Bella G, Veronese N, Barbagallo M (2021). Impact of mediterranean diet on chronic non-communicable diseases and longevity. Nutrients.

[10] Finicelli M, Di Salle A, Galderisi U, Peluso G (2022). The mediterranean diet: an update of the clinical trials. Nutrients.

[11] Malmir H, Saneei P, Larijani B, Esmaillzadeh A (2018). Adherence to Mediterranean diet in relation to bone mineral density and risk of fracture: a systematic review and meta-analysis of observational studies. Eur J Nutr.

[12] Dominguez LJ, Di Bella G, Veronese N, Barbagallo M (2021). Impact of mediterranean diet on chronic non-communicable diseases and longevity. Nutrients.

[13] Sotos-Prieto M, Bhupathiraju SN, Mattei J, Fung TT, Li Y, Pan A (2017). Association of changes in diet quality with total and cause-specific mortality. N Engl J Med.

[14] Shen Q, Xu Q, Li G, Ren L, Zhang Z, Zhang Y (2021). Joint effect of 25-hydroxyvitamin D and secondhand smoke exposure on hypertension in non-smoking women of childbearing age: NHANES 2007–2014. Environ Health.

[15] Looker AC, Orwoll ES, Johnston CC, Lindsay RL, Wahner HW, Dunn WL (1997). Prevalence of low femoral bone density in older U.S. adults from NHANES III. J Bone Miner Res.

[16] Fung TT, McCullough ML, Newby PK, Manson JE, Meigs JB, Rifai N (2005). Diet-quality scores and plasma concentrations of markers of inflammation and endothelial dysfunction. Am J Clin Nutr.

[17] Kim H, Kwon O (2019). Higher diet quality is associated with lower odds of low hand grip strength in the korean elderly population. Nutrients.

[18] Suzuki M, Urai S, Fukuoka H, Hirota Y, Yamamoto M, Okada Y (2022). Relation between the insulin lowering rate and changes in bone mineral density: analysis among subtypes of type 1 diabetes mellitus. J Diabetes Invest.

[19] Wang TK, Bolland MJ, van Pelt NC, Horne AM, Mason BH, Ames RW (2010). Relationships between vascular calcification, calcium metabolism, bone density, and fractures. J Bone Miner Res.

[20] Orwoll ES, Oviatt SK, Mann T (1990). The impact of osteophytic and vascular calcifications on vertebral mineral density measurements in men. J Clin Endocrinol Metab.

[21] Campos-Obando N, Castano-Betancourt MC, Oei L, Franco OH, Stricker BH, Brusselle GG (2014). Bone mineral density and chronic lung disease mortality: the rotterdam study. J Clin Endocrinol Metab.

[22] Lenchik L, Register TC, Hsu FC, Xu J, Smith SC, Carr JJ (2018). Bone mineral density of the radius predicts all-cause mortality in patients with type 2 diabetes: diabetes heart study. J Clin Densitom.

[23] Mathers JC (2006). Nutritional modulation of ageing: genomic and epigenetic approaches. Mech Ageing Dev.

[24] Schönenberger KA, Schüpfer AC, Gloy VL, Hasler P, Stanga Z, Kaegi-Braun N (2021). Effect of anti-inflammatory diets on pain in rheumatoid arthritis: a systematic review and meta-analysis. Nutrients.

