# Supplementary material for: Adherence to the Mediterranean diet regulates the association between osteopenia and the risk of all-cause mortality in general population
Source: J Health Popul Nutr. 2023 Oct 3;42:106. doi: 10.1186/s41043-023-00447-6 (PMC10546794; doi:10.1186/s41043-023-00447-6)
Supplement: Supplementary file 1 — Additional file 1. Table S1. The numbers and percentages of missing values. Table S2. Sensitivity analysis of missing values manipulation. [file 41043_2023_447_MOESM1_ESM.docx]

Supplementary Table 1 The numbers and percentages of missing values

| Variables | n | % |
| --- | --- | --- |
| Glucocorticoid use | 0 | 0.00% |
| Previous fracture | 2 | 0.04% |
| Marital status | 4 | 0.07% |
| Education | 6 | 0.11% |
| Weight | 12 | 0.22% |
| Height | 12 | 0.22% |
| Waist circumference | 43 | 0.79% |
| Drinking | 103 | 1.89% |
| Parents previous fracture | 112 | 2.05% |
| Total 25 Hydroxyvitamin D | 283 | 5.19% |
| PIR | 480 | 8.80% |

PIR: poverty-to-income ratio

Supplementary Table 2 Sensitivity analysis of missing values manipulation

|  | | Groups | |  | |
| --- | --- | --- | --- | --- | --- |
| Variables | Missing value, n (%) | Before manipulation | After manipulation | Statistics | *P* |
| Education, n (%) | 6 (0.11) |  |  | χ^2^=0.002 | 0.965 |
| High School of above |  | 2990 (54.90) | 2991 (54.86) |  |  |
| High School or below |  | 2456 (45.10) | 2461 (45.14) |  |  |
| Marital status, n (%) | 4 (0.07) |  |  | χ^2^=0.000 | 0.991 |
| Married |  | 3314 (60.83) | 3317 (60.84) |  |  |
| Other |  | 2134 (39.17) | 2135 (39.16) |  |  |
| PIR, ratio, M (Q_1_,Q_3_) | 480 (8.80) | 2.65(1.37,4.86) | 2.64(1.46,4.62) | Z=0.144 | 0.886 |
| Drinking, n (%) | 103 (1.89) |  |  | χ^2^=0.007 | 0.933 |
| No |  | 1449 (27.09) | 1473 (27.02) |  |  |
| Yes |  | 3900 (72.91) | 3979 (72.98) |  |  |
| Parents previous fracture, n (%) | 112 (2.05) |  |  | χ^2^=0.139 | 0.709 |
| No |  | 4764 (89.21) | 4876 (89.44) |  |  |
| Yes |  | 576 (10.79) | 576 (10.56) |  |  |
| Previous fracture, n (%) | 2 (0.04) |  |  | χ^2^=0.000 | 0.998 |
| No |  | 5369 (98.51) | 5371 (98.51) |  |  |
| Ye |  | 81 (1.49) | 81 (1.49) |  |  |
| Height, cm, Mean ± SD | 12 (0.22) | 167.17±9.90 | 167.16±9.89 | t=0.02 | 0.987 |
| Weight, kg, Mean ± SD | 12 (0.22) | 80.03±17.94 | 80.04±17.93 | t=-0.03 | 0.980 |
| Waist circumference, cm, Mean ± SD | 43 (0.79) | 100.32±13.68 | 100.35±13.72 | t=-0.09 | 0.930 |
| Total 25 Hydroxyvitamin D, mcg, M (Q_1_, Q_3_) | 283 (5.19) | 69.90(53.10,88.80) | 69.50(53.30,88.16) | Z=0.412 | 0.680 |
| Energy | 0 (0.00) |  |  |  |  |
| Calcium | 0 (0.00) |  |  |  |  |
| VitaminD | 0 (0.00) |  |  |  |  |
| Med | 0 (0.00) |  |  |  |  |
| Dietary supplements taken | 0 (0.00) |  |  |  |  |
| Time | 0 (0.00) |  |  |  |  |
| Osteopenia | 0 (0.00) |  |  |  |  |

PIR: poverty-to-income ratio
